# Genome Sequence of *Gordonia* Phage Emalyn

**DOI:** 10.1128/genomeA.00597-16

**Published:** 2016-08-11

**Authors:** Welkin H. Pope, Madeline J. Guido, Pragnya Iyengar, Jonathan T. Nigra, Matthew B. Serbin, Naomi S. Kasturiarachi, Catherine A. Pressimone, Johnathon G. Schiebel, Emily C. Furbee, Sarah R. Grubb, Marcie H. Warner, Matthew T. Montgomery, Rebecca A. Garlena, Daniel A. Russell, Deborah Jacobs-Sera, Graham F. Hatfull

**Affiliations:** Department of Biological Sciences, University of Pittsburgh, Pittsburgh, Pennsylvania, USA

## Abstract

Emalyn is a newly isolated bacteriophage of *Gordonia terrae* 3612 and has a double-stranded DNA genome 43,982 bp long with 67 predicted protein-encoding genes, 32 of which we can assign putative functions. Emalyn has a prolate capsid and has extensive nucleotide similarity with several previously sequenced phages.

## GENOME ANNOUNCEMENT

*Gordonia spp.* are actinomycetes found in soil and are associated with foaming in activated sludge of wastewater treatment plants (1). Bacteriophages of *Gordonia* hosts have been isolated and seventeen genomes have been sequenced (2–6) but whether the genetic diversity of *Gordonia* phages is as varied as the large collection of mycobacteriophages (7) remains ill-defined. The Science Education Alliance-Phage Hunters Advancing Genomics and Evolutionary Science (SEA-PHAGES) program provides a platform for exploring phages of actinobacterial hosts including phages of *Gordonia* (7, 8).

Phage Emalyn was isolated from a flower bed in Pittsburgh, Pennsylvania using *Gordonia terrae* 3612 as host. Plaques were recovered by direct plating of filtered soil extracts on lawns of *Gordonia*; phages were purified, amplified, and dsDNA was extracted. Emalyn forms relatively small plaques and can be readily propagated to high titer. Electron microscopy shows that Emalyn virions have a siphoviral morphotype with a long (300 nm) flexible tail, and a prolate head, 50 nm wide and 100 nm long.

The Emalyn genome was sequenced using an Illumina MiSeq with 140 bp single-end runs. Reads were assembled using Newbler to give a single major contig of 43,982 bp with 417-fold coverage. The viral genome has defined ends with 10 base 3′ extensions (5′-CGGTAGGCTT), and an overall G+C content of 61.2%, somewhat lower than that of *Gordonia terrae* (67.8%). Sixty-six predicted protein coding genes were identified using Glimmer and Genemark (9, 10), 31 of which could be assigned putative functions using BLASTp, HHpred, and Phamerator (11, 12). No tRNA genes were identified.

Emalyn has extensive nucleotide sequence similarity (87% identity) to the previously described *Gordonia* phage GTE2 (3) spanning 95% of their genome length. The two phages share the organization of rightwards-transcribed virion structure and assembly genes in their left arms, followed by a lysis cassette, and nonstructural genes transcribed both leftwards and rightwards in the right genome parts.

Curiously, the lysis cassette includes two separate genes encoding endolysin activities found as domains within a single protein in the mycobacteriophages (e.g., Myrna gp243); Emalyn gp19 and gp20 encode putative peptidase and glycoside hydrolase domains, respectively. Emalyn gp21 has two predicted transmembrane domains and is a holin candidate, supported by the finding that Emalyn gp21 homologues are found associated with endolysin genes in unrelated *Gordonia* phages. However, Emalyn gp22 has four predicted membrane spanning domains and may also be involved in lysis. Curiously, Emalyn encodes a putative lysin B protein (gp44), and homologues have been characterized previously only in the mycobacteriophages (13, 14). Furthermore, the lysin B gene (*44*) is atypically unlinked from the other lysis components, and sits in the right part of the genome downstream of a DNA polymerase I gene (*42*). The GTE2 homologue (gp37) is more distantly related to Emalyn gp44 (75% aa identity) than the DNA polymerase I proteins (95.6% aa identity). Other Emalyn-encoded proteins of note are RecA (gp57), two helicases (gp41, gp52), ThyX (gp46), and a kinase (gp50).

### Accession number(s).

The Emalyn genome is available from GenBank under accession number KU963260.
